# In Situ N, O Co-Doped Nanoporous Carbon Derived from Mixed Egg and Rice Waste as Green Supercapacitor

**DOI:** 10.3390/molecules28186543

**Published:** 2023-09-09

**Authors:** Shumeng Qin, Peiliang Liu, Jieni Wang, Chenxiao Liu, Shuqin Zhang, Yijun Tian, Fangfang Zhang, Lin Wang, Leichang Cao, Jinglai Zhang, Shicheng Zhang

**Affiliations:** 1 Miami College, Henan University, Kaifeng 475004, China; qsm9452@henu.edu.cn (S.Q.); lpl0672@henu.edu.cn (P.L.); jieniwang@126.com (J.W.); lcxdxyyx@henu.edu.cn (C.L.); zhangshuqin@henu.edu.cn (S.Z.); 104753200976@henu.edu.cn (Y.T.); zhff@henu.edu.cn (F.Z.); wanglin@henu.edu.cn (L.W.); 2 College of Chemistry and Molecular Sciences, Henan University, Kaifeng 475004, China; zhangjinglai@henu.edu.cn; 3 Shanghai Key Laboratory of Atmospheric Particle Pollution and Prevention (LAP3), Department of Environmental Science and Engineering, Fudan University, Shanghai 200433, China; zhangsc@fudan.edu.cn

**Keywords:** N, O co-doped, porous carbon, egg waste, rice waste, supercapacitor

## Abstract

The conversion of nitrogen–oxygen-rich biomass wastes into heteroatomic co-doped nanostructured carbons used as energy storage materials has received widespread attention. In this study, an in situ nitrogen–oxygen co-doped porous carbon was prepared for supercapacitor applications via a two-step method of pre-carbonization and pyrolytic activation using mixed egg yolk/white and rice waste. The optimal sample (YPAC-1) was found to have a 3D honeycomb structure composed of abundant micropores and mesopores with a high specific surface area of 1572.1 m^2^ g^−1^, which provided abundant storage space and a wide transport path for electrolyte ions. Notably, the specific capacitance of the constructed three-electrode system was as high as 446.22 F g^−1^ at a current density of 1 A g^−1^ and remained above 50% at 10 A g^−1^. The capacitance retention was 82.26% after up to 10,000 cycles. The symmetrical capacitor based on YPAC-1 with a two-electrode structure exhibited an energy density of 8.3 Wh kg^−1^ when the power density was 136 W kg^−1^. These results indicate that porous carbon materials prepared from mixed protein and carbohydrate waste have promising applications in the field of supercapacitors.

## 1. Introduction

The rapid global economic development and increasing population have gradually expanded the demand for energy. The finite nature of fossil energy and environmental pollution are also attracting growing concern. Therefore, the search for new and renewable energy storage technologies has become an important issue worldwide [1]. Supercapacitors are attracting attention due to their high energy density, long cycle life, and environmental friendliness. Compared with traditional energy storage batteries, they can be charged and discharged quickly in a short period of time and are now widely used in applications that require high energy and power densities [2].

Based on the principle of energy storage, supercapacitors can be divided into double-electric layer capacitors (EDLCs) and pseudocapacitors [3]. The energy storage principle in EDLCs is electrostatic, which stores energy via the adsorption/desorption of charged ions at the electrolyte interface on the surface of the electrode material [4]. Therefore, EDLCs require a large specific surface area and abundant pores to enhance their electrochemical performance. Compared to metal oxides and conductive polymers, carbon materials have higher specific surface area, good conductivity, stability, and are more cost-effective and environmentally friendly [5]. Their electrochemical performance can be significantly improved by modulating the microstructure of carbon materials, such as pore size and surface functional groups. Thus, carbon materials are often used to prepare EDLCs. Meanwhile, pseudocapacitors mainly store energy through the reversible Faraday redox reactions of electroactive materials, such as conducting polymers and transition metal oxides, on the electrode surface [6,7,8]. However, the volume of conducting polymers is prone to change during the charging and discharging process, which destroys the microstructure of molecular chains [9,10]. Therefore, scientists often compound conducting polymers with carbon materials to alleviate the problem of volume expansion. For example, Niu et al. [11] prepared coral-like hollow poly (3,4-ethylenedioxythiophene) nanotubes for supercapacitor applications via electrochemical polymerization assisted by carbon fiber cloth templates. The introduction of carbon fibers not only provides excellent tensile strength but also enables uniform load distribution and protects the fibers from external damage [12]. Carbon materials are important candidates in the field of supercapacitors, and the development of carbon electrode materials with better structures is the key to improving the performance of supercapacitors.

Carbon electrode materials ranging from 0D to 3D nanostructures have been explored to obtain better carbon electrode materials, such as graphene, carbon fibers, activated carbon, and porous carbon derived from metal–organic frameworks [13,14,15,16]. Although these carbon materials have good framework structures and are widely used in supercapacitors, their surface chemistry and electrochemical activity still need further optimization [17]. Doping with heteroatoms is considered to be one of the most effective ways to enhance the electrochemical performance of porous carbon electrode materials. Among them, the introduction of N can effectively improve the electronic structure and surface chemistry of porous carbon materials by replacing some of the C on the graphite lattice to form positively charged hybrid orbitals [18,19]. This method improves the electrical conductivity and reaction rate of the electrode. Compared with additional nitrogen sources such as melamine, urea, and ammonia [20,21,22], choosing nitrogen-rich biomass waste as a porous carbon precursor is more beneficial to the green environment and does not cause secondary pollution to the environment [23]. Furthermore, in situ nitrogen doping is more stable compared to introducing additional nitrogen sources and can effectively reduce the loss of nitrogen content during the doping process [24]. In industrial production, large quantities of industrial-grade eggs containing special additives are used to extract purely natural chemicals such as lecithin and egg whites for use in antibacterial products and cosmetics [25]. This process generated a large amount of inedible egg waste. Eggs are rich in protein, which provides an abundant source of nitrogen-containing groups (-NH_2_). This inherent nitrogen content allows for in situ nitrogen doping during the pyrolysis process, eliminating the requirement for additional nitrogen sources [26]. However, the low carbon content of eggs is not conducive to the formation of a carbon skeleton after carbonization. Thus, mixing them with carbon-rich waste biomass can increase their carbon content, which facilitates the formation of the carbon skeleton and improves their electrochemical performance. Rice waste, as a common food waste, is mainly composed of starch, which is a polymeric carbohydrate. It is often disposed of via incineration without reasonable utilization [27]. Therefore, recycling rice and egg waste to produce porous carbon through in situ nitrogen–oxygen co-doping and applying it in supercapacitors can open up new pathways for the renewable utilization of solid waste [28]. Furthermore, they can also serve as typical food waste materials to explore their potential as ideal precursors for carbon-based electrode materials.

In this study, egg yolks and whites were individually mixed with rice waste as ideal precursors for the synthesis of porous carbon electrode materials. An in situ nitrogen–oxygen co-doped porous carbon material was prepared via a two-step method of pre-carbonization and pyrolytic activation. The effect of the mixture ratio of egg yolk/white and rice on the electrochemical properties (e.g., GCD, CV, EIS, and cycling performance) and porous carbon structure (e.g., S_BET_, pore volume, and pore size) was investigated. The optimal sample exhibited a specific capacitance of 446.22 F g^−1^ (1 A g^−1^, 6 M KOH), and the capacitance retention rate was 82.26% after 10,000 cycles. This work demonstrated that the combination of typical proteins (such as egg) with carbohydrates (such as rice) had excellent supercapacitor performance. It also suggested the potential benefits of using mixed food waste as a precursor for the preparation of porous carbon materials. These findings provided a new perspective for the subsequent treatment of mixed food waste.

## 2. Results and Discussion

### 2.1. Morphology and Structure Analyses of N, O Co-Doped Porous Carbon

**Figure 1:**
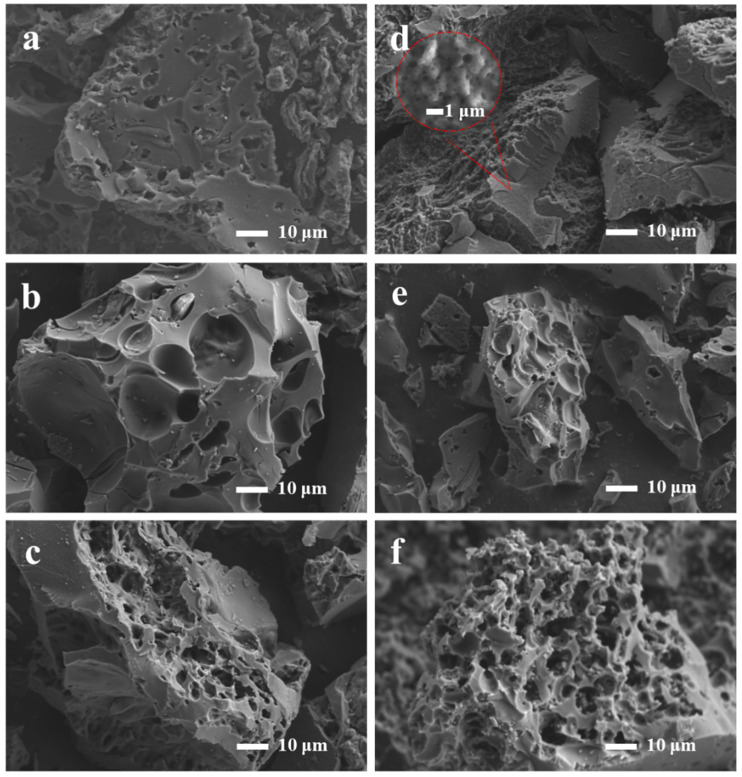
Scanning electron micrographs of (**a**) WPAC-1, (**b**) WPAC-2, (**c**) WPAC-0, (**d**) YPAC-1, (**e**) YPAC-2, and (**f**) YPAC-0.

Figure 1 shows the SEM images of all the prepared samples. All of the samples exhibited abundant pore structure, and the heat-treated biomass-based derivative porous carbon transformed from a bulk structure to a 3D honeycomb structure as the percentage of egg yolk/white precursors increased. As shown in Figure 1d, YPAC-1 exhibited coarse micron-sized pores, and more abundant pores were observed with increasing magnification. This finding indicates that YPAC-1 had a graded porous structure. The main reason for the coexistence of this unique morphology with various-sized pores is the release of a large number of gases (e.g., CO_x_ and H_2_) during the pyrolysis of collagen [29]. The 3D honeycomb porous carbon material with rich porosity prepared from egg yolk/white is a promising candidate for supercapacitor applications; this material not only facilitates effective electrochemical contact between electroactive materials and electrolytes but also provides abundant storage space and a wide transport path for electrolyte ions [30].

**Figure 2:**
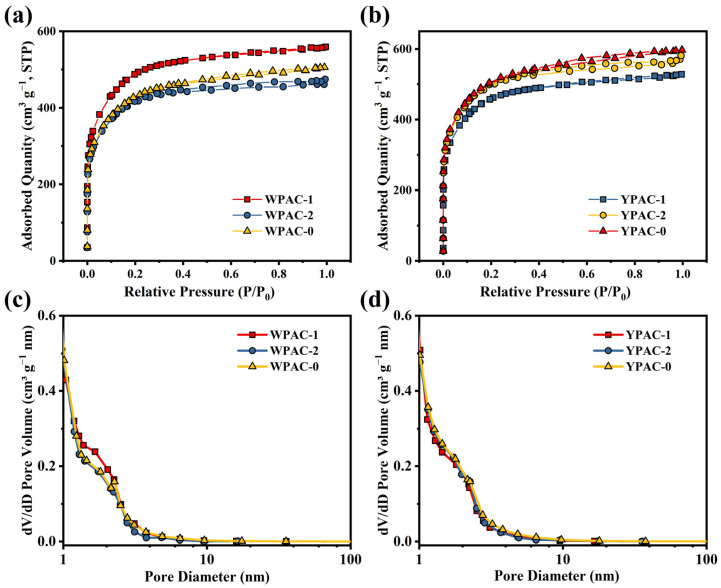
(**a**,**b**) N_2_ adsorption–desorption isotherms and (**c**,**d**) pore size distribution of nitrogen-doped porous carbon prepared based on egg yolk/white.

Figure 2 shows the N_2_ adsorption–desorption curves and pore size distribution of the samples. The N_2_ adsorption–desorption isotherms of WPAC-1 and YPAC-1 are observed in Figure 2a,b were consistent with type I isotherms (IUPAC classification criteria), which indicates that they were dominated by narrow micropores (<1 nm) [31]. However, the N_2_ adsorption–desorption curves for WPAC-2, WPAC-0, YPAC-2, and YPAC-0 exhibited adsorption hysteresis loops, which is a characteristic feature of type IV isotherms; this result indicates the coexistence of micropores and mesopores [32]. Additionally, under high pressure conditions (0.95 < P/P_0_ < 1.0), the N_2_ adsorption–desorption isotherm shows a slight increase, indicating the presence of a small number of macropores in the material [33]. Secondly, the pore size distribution of the samples was calculated in accordance with the N_2_ adsorption–desorption isotherm using NLDFT (Figure 2c,d). The pore sizes of all samples were mainly distributed between 0 and 10 nm, which showed that all carbon materials were rich in micropores (<2 nm) and mesopores (2–50 nm). The specific surface area and pore parameters of different samples are shown in Table 1. It is not difficult to observe that the specific surface areas of all samples are within the range of 1400–1700 m^2^ g^−1^, and the percentage of micropores ranges from 41% to 54%. This is mainly due to the consistent activation conditions of all samples, resulting in similar specific surface areas and proportions of micropores. In summary, these samples possess excellent pore structures, which is beneficial for the enhancement of electrochemical performance. Micropores inside have multiple twisted and narrow channels, which can shorten the diffusion path of ions in electrode materials and increase the migration rate of ions in the electrode. Mesopores can allow ions and solutions to rapidly permeate the surface of the electrode [34]. A small number of macropores could reduce the resistance to electrolyte transfer and facilitate ion or reactant diffusion [35,36].

**Figure 3:**
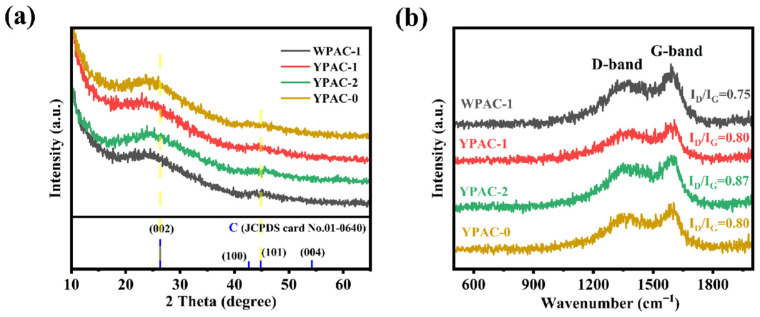
(**a**) XRD pattern and (**b**) Raman spectra of WPAC-1, YPAC-0, YPAC-1, and YPAC-2.

Figure 3 and Figure S1 show the XRD pattern and Raman spectra of the nitrogen-doped porous carbon prepared based on egg yolk/white. As shown in Figure 3a and Figure S1a, all samples had two broad diffraction peaks at 26.5° and 44.5°. By comparing with the standard card of graphite (JCPDS card No.01-0640), it can be observed that the peak at 26.5° corresponds to the (002) non-crystalline carbon of graphite, and the weak diffraction peak at 44.5° corresponds to (101), further indicating its degree of graphitization [37]. Therefore, these samples have a low degree of graphitization and exhibit a non-crystalline carbon structure, and this amorphous structure could promote the transport of charged ions [38]. The Raman spectra of all samples are shown in Figure 3b and Figure S1b. Two characteristic peaks were observed at 1360 and 1590 cm^−1^. The two peaks are typical of the D-band and G-band, respectively. The I_D_/I_G_ values of the nitrogen-doped porous carbon materials prepared based on egg white were calculated to be 0.75, 0.89, and 0.85. The I_D_/I_G_ values of nitrogen-doped porous carbon materials prepared based on egg yolk were 0.80, 0.87, and 0.80. The effect of adding egg yolk/white on the I_D_/I_G_ was investigated under the same activation conditions for all samples. The results showed that the addition of protein (egg yolk/white as the main nitrogen source) had a weak effect on the I_D_/I_G_, which means that the graphitization of porous carbon materials was insignificantly affected by the amount of nitrogen-containing biomass added.

Table 2 shows the results of elemental analysis of all samples. The highest N content (4.25% and 3.54% for WPAC-1 and YPAC-1, respectively) was obtained when egg yolk/white was mixed with rice waste in equal proportions as precursors. The sample yield ranges from 33.77% to 51.11%, demonstrating an essentially consistent trend with the variation in nitrogen content. However, with the increase in protein (egg yolk/white) dosage, the N content of WPAC-2, WPAC-0, YPAC-2, and YPAC-0 decreased significantly after the 600 °C pyrolysis activation. This result is probably due to the fact that a more stable nitrogen-containing carbon skeleton could be formed by combining with the carbon-containing compounds provided by rice flour when the protein pyrolysis was converted to other nitrogen-containing compounds. Therefore, the addition of a moderate amount of rice flour (carbon source) could play a role in nitrogen fixation and reduce the loss of nitrogen. Furthermore, the O content in the nitrogen-doped porous carbon prepared based on egg yolk/white decreased gradually with the addition of rice waste. This finding is probably due to the fact that the O content of the egg yolk/white itself was small and the high-temperature pyrolysis would further increase the O loss. However, the addition of typical carbohydrates (rice waste) could provide a certain amount of O to reduce the O loss during high-temperature pyrolysis.

### 2.2. Chemical Composition Analysis of Porous Carbon

**Figure 4:**
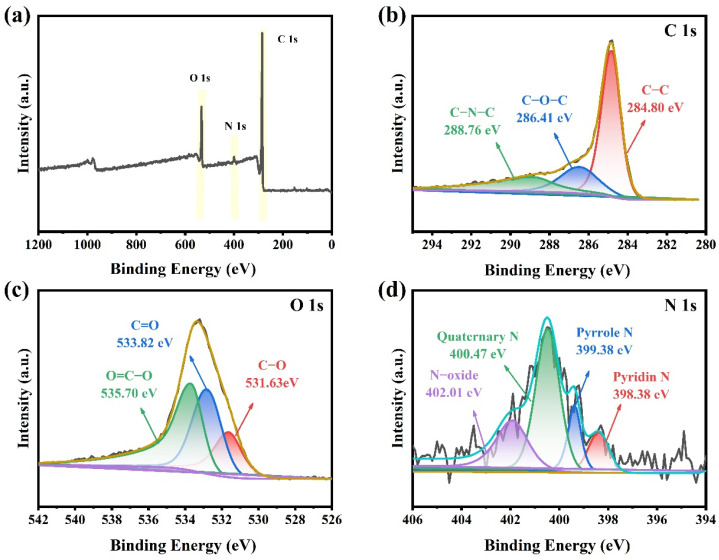
(**a**) XPS full spectrum and (**b**–**d**) C 1s, O 1s, and N 1s high-resolution spectra of YPAC-1.

The chemical composition of YPAC-1 was further analyzed with XPS (Figure 4). The XPS spectra of Figure 4a show three different peaks of C (283.67 eV), N (399.22 eV), and O (531.11 eV) with content percentages of 87.76%, 2.81%, and 10.14%, respectively. The C 1s peaks at 284.80, 288.76, and 286.41 eV corresponds to C–C, C–N–C, and C–O–C, respectively (Figure 4b). As shown in Figure 4d, N 1s could be deconvoluted into N-6 (398.38 eV, pyridine N), N-5 (399.38 eV, pyrrole N), N-Q (400.47 eV, quaternary N), and N-X (402.01 eV, N-oxide). N-5 and N-6 facilitate the formation of surface and edge defects in carbon materials, as well as an increase in electrochemical reaction sites [39]. The lone pair electrons on their nitrogen atoms can participate in electron transfer and charge storage reactions, known as the Faradaic reaction (Equations (1) and (2)) [40]. These lone pair electrons interact with ions in the electrolyte (KOH), promoting the adsorption and dissolution of electrolyte ions on the electrode surface. In the Faradaic reaction, with a change in potential, the ions in the electrolyte undergo oxidation–reduction reactions on the electrode surface, forming an electrical double layer and generating pseudocapacitance. This provides a potential for high energy density and fast charge–discharge capabilities in capacitors.

(1) $$>\;\mathrm{CH}\text{-}NH_{2}+2OH^{-}\;↔\;>\;C\text{=}\mathrm{NH}+2H_{2}+2e^{-}$$

(2) $$>\;C\text{-}NH_{2}+2OH^{-}\;↔\;>\;C\text{-}\mathrm{NHOH}+H_{2}O+2e^{-}$$

N-Q and N-X in the carbon skeleton could provide a positive charge, change the charge density, and alleviate the resistance to charge migration, which improves the electrical conductivity [41]. O 1s in Figure 4d could be deconvoluted into three peaks, namely, C=O (533.82 eV), C–O (531.63 eV), and O=C–O (535.70 eV), respectively. Oxygen-containing groups can improve the wettability of carbon materials to aqueous electrolytes and introduce Faraday pseudocapacitance to enhance electrochemical properties [42]. N, O co-doped porous carbon as an electrode material had significant electron-donor properties and high charge mobility, which could induce electron-donor properties and provide high surface energy and surface reactivity activity during electron transfer. These conditions could improve the surface wettability of the electrode material, which was beneficial to increase the contact area between the electrolyte and the electrode material [43]. The abovementioned analysis demonstrated that the mixture of egg yolk/white and rice waste as the precursor through pyrolysis pre-carbonization and KOH activation coupling process successfully doped nitrogen into the carbon skeleton.

### 2.3. Electrochemical Performance

**Figure 5:**
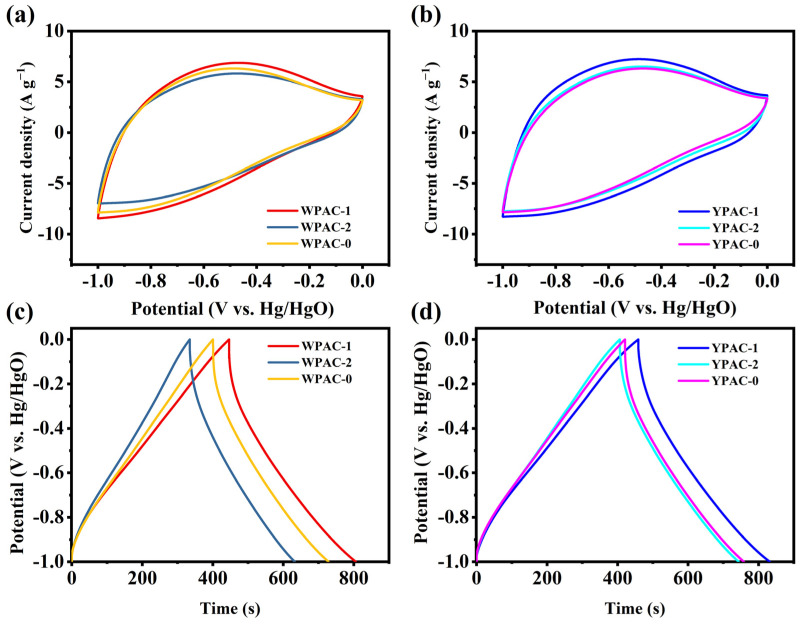
(**a**,**b**) CV curves of nitrogen-doped porous carbon prepared based on egg yolk/white at a sweep rate of 20 mV s^−1^. (**c**,**d**) GCD curves of nitrogen-doped porous carbon prepared based on egg yolk/white at a current density of 1 A g^−1^.

In a three-electrode system, nitrogen-doped porous carbon based on egg yolk/white was tested for supercapacitor performance using a 6 M KOH electrolyte. The CV curves obtained at a scan rate of 20 mV s^−1^ for the egg yolk/white nitrogen-doped porous carbon electrodes are shown in Figure 5a,b. Weak humps were observed in the current range of −0.3 V to −0.7 V due to Faraday reactions generated by pyrrole N and pyridine N. Pyrrole N provided additional reversible pseudocapacitance, which gave the electrode excellent electrochemical performance. The constant current charge–discharge curves of the nitrogen-doped porous carbon prepared based on egg yolk/white were triangular (Figure 5c,d), but the GCD curves were not strictly symmetric due to the presence of pseudocapacitance [44]. YPAC-1 had the longest discharge time and the highest specific capacitance among all samples. As described in the previous section on pore characteristics and XPS analysis results, the combination of high specific surface area enhancing the viability of the charge accumulation surface, multilevel pore structure accelerating ion transport and diffusion, and N doping changing the characteristics of carbon electron donor/acceptor were the main factors affecting the performance of the nitrogen-doped porous carbon supercapacitors based on egg yolk/white [45].

**Figure 6:**
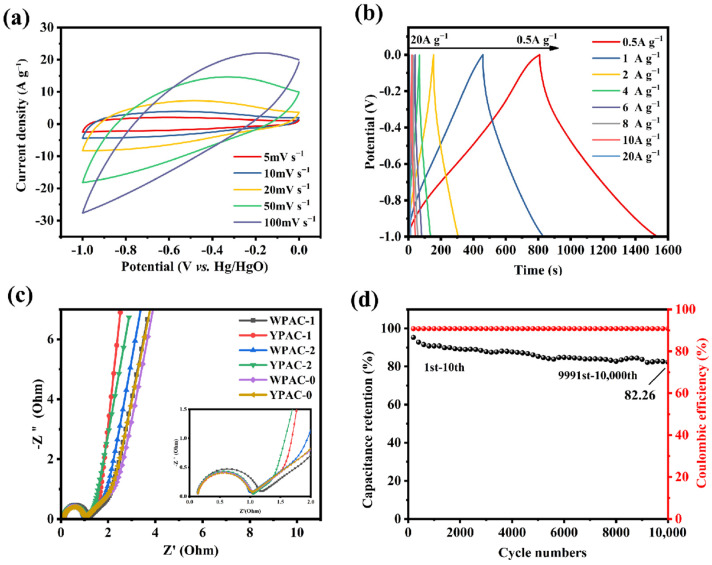
(**a**) CV curves of YPAC-1 at different scan rates. (**b**) GCD curves of YPAC-1 at different current densities. (**c**) impedance profiles of all samples. (**d**) Cycling stability and Coulomb efficiency diagram of YPAC-1 in the three-electrode system.

Electrochemical tests such as CV and GCD were performed (Figure 6a,b) to investigate the electrochemical performance of YPAC-1 as a supercapacitor electrode material in the three-electrode system. The CV curves of YPAC-1 at low scan rates (5–20 mV s^−1^) exhibited a rectangular-like shape, which indicates that EDLC was dominant in this sample [46]. The CV curve exhibited a significant distortion with the increase in scan rates, and this distortion is due to the restriction of ion diffusion at high scan rates [47]. In this study, there is no need to add extra nitrogen sources during the preparation process. Consequently, the steps for nitrogen doping are eliminated, making the preparation process more convenient and cost-effective. Additionally, the in situ nitrogen doping helps minimize nitrogen loss, leading to enhanced nitrogen retention in the material [24]. Moreover, YPAC-1 exhibited outstanding specific capacitance properties compared to porous carbons derived from previous waste biomass (Table 3). Figure S2 summarizes the specific capacitance of YPAC-1 at different current densities. At a current density of 1 A g^−1^, it achieved a specific capacitance of 446.22 F g^−1^, significantly higher than that of its counterparts. Remarkably, even at a high current density of 20 A g^−1^, YPAC-1 maintained a specific capacitance of 136.55 F g^−1^. Figure 6c displays the EIS of the nitrogen-doped porous carbon prepared from egg yolk/white. The near-vertical Nyquist plots observed in the low-frequency region indicate the ideal capacitive behavior of all samples due to the mesoporous and macroporous structure of the material [31,47]. This structure shortened the ion diffusion distance and provided more ion diffusion channels, which enhanced the capacitive performance of the electrode material [48]. As evident in the mid-high-frequency region (inset in Figure 6c), all samples showed a transition from the 45° Warburg region to a semicircle, which could be attributed to ion diffusion and charge transfer. The slope of the straight line in the low-frequency region reflected the equivalent series resistance (R_s_) of the material, and the R_s_ of YPAC-1 was the smallest among all samples (R_s_ = 2.43 Ω). In the high-frequency region, the charge transfer resistances (R_ct_) value of YPAC-1 was also smaller (R_ct_ = 0.73 Ω), which means that it had a smaller load transfer resistance. Moreover, the R_s_ of YPAC-1 was 0.107 Ω, which suggests that the mass transfer and load transfer resistance of this sample were relatively small. YPAC-1 also underwent 10 A g^−1^ constant-current charge/discharge cycles. The charge/discharge performance of the material started to decrease after 1000 cycles. This finding is probably due to the lower pseudocapacitance provided by the heteroatoms, which led to a decrease in capacitor performance. However, the decrease in charge/discharge performance of YPAC-1 slowed down as the number of cycles increased. The capacitance retention of the device was 82.26% after 10,000 cycles (Figure 6d). This result is due to the combined synergistic effect of N doping, a higher specific surface area (1572.1 cm^2^ g^−1^), and a multistage porous structure. These conditions maintained the good Coulombic efficiency of the device even during cycling, which enabled it to exhibit better durability [49].

In order to further analyze the capacitance properties of YPAC-1, b-value graph was used to study the power-law relationship between the peak current (*i*) and the scanning rate (*v*), expressed as *i = av^b^*. The b-value can be used to determine the charge storage behavior, where values between 0 and 0.5 indicate diffusion-controlled behavior, defined as battery-type materials. When the b value is 0.5–1.0, it proves that the electrode material is capacitive, which presents as super capacitance grading material between 0.5–0.8, and a supercapacitor property is displayed at 0.8–1.0 [44]. As shown in Figure S3, the b-value of YPAC-1 is 0.77, indicating it possesses both battery and capacitor characteristics.

**Figure 7:**
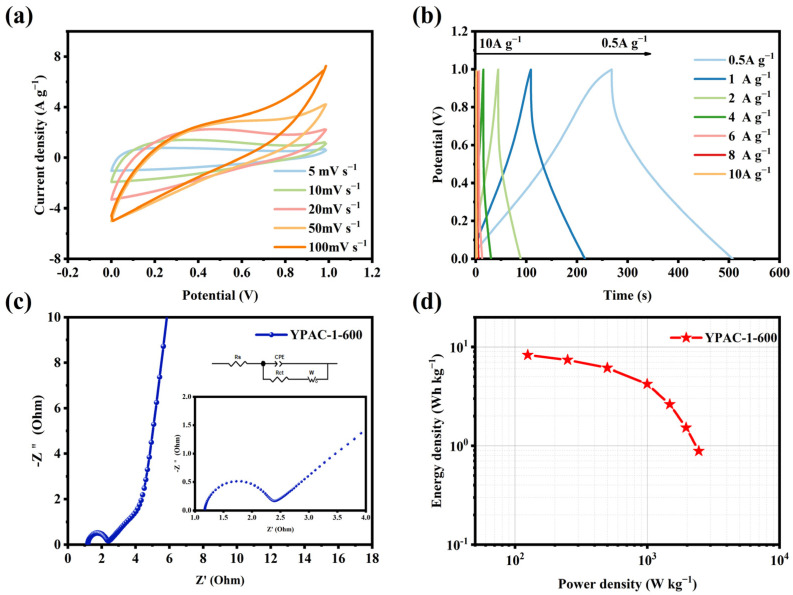
Electrochemical performance of YPAC-1-600 based supercapacitor tested in a two-electrode configuration in an aqueous electrolyte of 6 M KOH. (**a**) CV curve (5–100 mV s^−1^). (**b**) GCD curve (1–10 A g^−1^). (**c**) Impedance profile. (**d**) Ragone plot.

The symmetric supercapacitor was assembled using two electrode sheets prepared from YPAC-1 and tested in a 6 M KOH electrolyte. Figure 7a illustrates the CV curves of YPAC-1 at scan rates in the range of 5–100 mV s^−1^. The curves show an approximately rectangular shape, which suggests that the material had EDLC performance. As observed in Figure 7b, the YPAC-1 two-electrode system exhibited symmetrical triangular curves at 0.5–10 A g^−1^. This result means that the nitrogen-doped porous carbon prepared based on egg yolk delivered excellent reversibility [52]. The electrochemical impedance spectrum of the two-electrode system was observed at low frequencies (Figure 7c), which showed a straight line nearly parallel to Z″. This finding means that the YPAC-1 multilayer pore structure provided more diffusion channels. The small Warburg portion at the intermediate frequency and the small semicircle in the high-frequency region (with a diameter of 4.43 Ω) corresponded to fast ions diffusing to the porous carbon electrode and a lower charge transfer resistance, respectively. Furthermore, the real axis intercept (at 0.17 Ω) indicated low contact resistance and good conductivity, which further demonstrated the excellent capacitive performance of the YPAC-1 electrode. As shown in Figure 7d, the device exhibited an energy density of 8.3 Wh kg^−1^ at power densities of 136 and 1.58 Wh kg^−1^ under a high power density of 2472.6 W kg^−1^.

## 3. Materials and Methods

### 3.1. Materials and Chemicals

The eggs and rice waste were purchased from the South Court Restaurant of Henan University, and the egg whites and yolks were separated manually from the eggs (Kaifeng, Henan, China). The chemical reagents used in this study included anhydrous potassium carbonate (KOH, 85%) purchased from Tianjin Kermel Chemical Reagent Co., Ltd. (Tianjin, China) and hydrochloric acid (HCl, 38%) procured from Sinopharm Chemical Reagent Co., Ltd. (Shanghai, China).

### 3.2. Synthesis of N, O Co-Doped Porous Carbon

**Figure 8:**
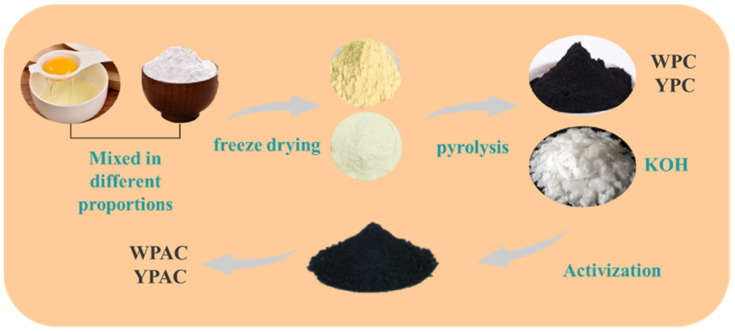
Schematic flowchart of WPC/YPC and WPAC/YPAC sample preparation.

The preparation process of in situ nitrogen-doped carbon-based materials based on egg yolk/white and waste rice is shown in Figure 8. The egg yolk/white and waste rice powder were mixed in a beaker with mass ratios of 1:1, 1:1.5, and 1:0. Then, they were placed in a holding tank with liquid nitrogen for rapid freezing. Thereafter, they were transferred to a freeze dryer after complete freezing and kept for 72 h to obtain the egg yolk/white precursor. The egg yolk/white precursors were added to corundum boats and heated up to 600 °C in a tube furnace at 2 °C min^−1^ under a nitrogen atmosphere and maintained for 2 h. Next, the solid products were washed three times each with anhydrous ethanol and deionized water, and they were dried overnight in a vacuum drying oven at 80 °C. This process produced pre-carbonized egg white biochars, which were labeled as WPC-1, WPC-2, and WPC-0, and egg yolk biochars, which were designated as YPC-1, YPC-2, and YPC-0. The abovementioned biochar and KOH were weighed at a mass ratio of 1:4 and ground in an agate mortar. The ground mixture was added to a nickel crucible, followed by pyrolytic activation in a tube furnace under a nitrogen atmosphere (N_2_ flow rate of 25 mL min^−1^). The temperature was increased to 600 °C at a rate of 2 °C min^−1^ and maintained for 2 h. After cooling to room temperature, the obtained samples were washed three times with 10 wt% HCl and finally washed with deionized water to neutralize. The solid products were dried overnight in a vacuum drying oven at 80 °C. The resulting nitrogen-doped porous carbon samples derived from egg white were named WPAC-1, WPAC-2, and WPAC-0, and those derived from egg yolk were named YPAC-1, YPAC-2, and YPAC-0.

### 3.3. Material Characterization

Scanning electron micrographs of sample morphology were obtained via field-emission scanning electron microscopy (SEM, Carl Zeiss, Oberkochen, UK). The elemental composition of the samples was analyzed using a Vario EL cube (Elementar, Frankfurt, Germany). The X-ray photoelectron spectra (XPS) of the samples were obtained using AXIS ULTRA (Kratos, Manchester, UK). X-ray diffractograms from 5° to 80° were acquired using a powder X-ray diffractometer (XRD, Bruker D8 Advance, Bruker AXS, Saarbrucken, Germany). The specific surface area and pore characteristics of the samples were analyzed by N_2_ adsorption and desorption data at −196 °C using Micromeritics ASAP 2020 M (Micromeritics, Norcross, GA, USA). Raman spectra were obtained using a laser micro-Raman spectrometer (Renishaw, Gloucestershire, UK) to characterize the lattice structure in carbon materials.

### 3.4. Electrochemical Measurements

First, the electrode material needed to be prepared. In the three-electrode system, carbon sample, carbon black, and polytetrafluoroethylene binder were mixed in the weight ratio of 8:1:1. Then, an appropriate amount of anhydrous ethanol was added and ground thoroughly in a mortar. The ground composite was subsequently coated on a 1 × 1 cm^2^ sheet of nickel foam. The obtained samples were compressed by a press at 15 MPa for 2 min and collected to obtain the target working electrodes.

The electrochemical measurements were conducted using an electrochemical workstation (CHI760E, Shanghai, China). In the three-electrode system, 6 M KOH solution was used as the electrolyte, and a Pt electrode and HgO/Hg electrode were used as the counter and reference electrodes, respectively. In the two-electrode system, two identical electrode materials were used as positive and negative electrodes, and a polypropylene diaphragm was selected for the assembly of the symmetric capacitor. The electrochemical tests involved include cyclic voltammetry (CV), constant current charge/discharge (GCD), electrochemical impedance spectroscopy (EIS), and cycling performance tests.

In the three-electrode system, the specific capacitance *C*_1_ (F g^−1^) of the GCD can be calculated with Equation (3):

(3) $$C_{1}=I\times \Delta t/m\times \Delta V$$

where *I* (A) is the discharge current, Δ*t* (s) is the discharge time, *m* (g) is the mass of the electrode material, and Δ*V* (V) is the potential window.

In the two-electrode system, the specific capacitance of the assembled symmetrical capacitor *C*_2_ (F g^−1^) was calculated from Equation (4):

(4) $$C_{2}=2\times I\times \Delta t/m\times \Delta V$$

where *I* (A) is the discharge current, Δ*t* (s) is the discharge time, *m* (g) is the total mass of the electroactive material, and Δ*V* (V) is the potential window.

The energy density *E* (Wh kg^−1^) and power density *P* (W kg^−1^) were calculated with Equations (5) and (6), respectively:

(5) $$E=\frac{1}{2\times 3.6}\times C_{1}\times {\Delta V}^{2}$$

(6) $$P=\frac{E}{\Delta t}\times 3600$$

where Δ*V* (V) is the electric potential and Δ*t* (s) is the discharge time.

## 4. Conclusions

Egg (as a typical protein) and rice waste (as a typical carbohydrate) were used as the natural nitrogen and carbon sources, respectively, to prepare in situ nitrogen-doped porous carbon via pre-carbonization coupled with pyrolytic activation for use as a green supercapacitor. The effect of rice flour addition on the microstructure and supercapacitor performance of the material was investigated. The results showed that, when the mass ratio of egg yolk/white to waste rice powder was 1:1, the material had a unique honeycomb-like porous morphology with a high specific surface area of 1572.1 m^2^ g^−1^. These porous properties not only increased the contact with the electrolyte but also facilitated the rapid diffusion of ions. Notably, the introduction of N facilitated the formation of pseudocapacitance, which increased not only the electrical conductivity but also the surface wettability. The specific capacity of YPAC-1 was 446.22 F g^−1^ at 1 A g^−1^, and the capacitance retention rate was 82.26% after 10,000 cycles. The device exhibited an energy density of 8.3 Wh kg^−1^ at a power density of 136 W kg^−1^.

## Figures and Tables

**Table 1:** Pore parameters of nitrogen-doped porous carbon based on egg yolk/white.

Sample	S_BET_ ^1^	S_mic_ ^2^	V_mic_ ^3^	V_t_ ^4^	V_mic_/V_t_
	(m^2^ g^−1^)	(m^2^ g^−1^)	(cm^3^ g^−1^)	(cm^3^ g^−1^)	(%)
WPAC-1	1666.4	906.5	0.4081	0.8651	47.17%
WPAC-2	1414.8	831.3	0.3753	0.7342	51.12%
WPAC-0	1466.1	782.1	0.3521	0.7822	45.01%
YPAC-1	1572.1	978.9	0.4412	0.8164	54.04%
YPAC-2	1691.0	809.4	0.3684	0.8985	41.00%
YPAC-0	1788.4	1111.1	0.47	0.9235	50.89%

^1^ Specific surface area was calculated using the BET method at P/P_0_ = 0.005–0.05; ^2^ Surface area of micropores (<2 nm); ^3^ Pore volume of micropores (<2 nm) obtained from t-plot method; ^4^ Total pore volume at P/P_0_ = 0.99.

**Table 2:** Elemental analysis of nitrogen-doped porous carbon based on egg yolk/white.

Sample	C (%)	H (%)	N (%)	O (%)	H/C	O/C	(O+N)/C	Yield (%)
WPAC-1	67.63	2.76	4.25	25.37	0.041	0.375	0.438	43.21%
WPAC-2	57.77	4.39	1.50	36.35	0.076	0.629	0.655	51.11%
WPAC-0	82.76	1.86	1.78	13.61	0.023	0.164	0.186	39.97%
YPAC-1	63.63	3.25	3.54	29.58	0.051	0.465	0.521	42.31%
YPAC-2	70.04	2.35	1.51	26.11	0.0336	0.3727	0.3942	49.56%
YPAC-0	87.69	2.63	1.07	8.61	0.0300	0.0982	0.1104	33.77%

**Table 3:** Comparison of various biowaste derived porous carbon as an electrode material.

Precursors	Measurements Done at (A/g)	Specific Capacitance (F g^−1^)	Electrolyte	Reference
Egg yolk + waste rice	1	446.22	6 M KOH	This work
Albizia flowers	0.5	406	6 M KOH	[4]
American Poplar fruit waste	1	423	6 M KOH	[50]
bamboo chips	1	208	6 M KOH	[22]
Orange peels	0.5	352	6 M KOH	[43]
Turbinaria Conoides	1	416	1 M H_2_SO_4_	[51]

## Data Availability

Data sharing not applicable.

## Supplementary Materials

The following supporting information can be downloaded at: https://www.mdpi.com/article/10.3390/molecules28186543/s1, Figure S1: (a) XRD pattern and (b) Raman spectra of WPAC-0, WPAC-1, and WPAC-2. Figure S2: The specific capacitance of YPAC-1 at 0.5–20 A g^−1^. Figure S3: Relationship between peak current and scan rates at scan rates of 5–100 mV s^−1^.

## Sample Availability

Samples of the compounds are available from the authors upon request.
