# Supplementary material for: In Situ N, O Co-Doped Nanoporous Carbon Derived from Mixed Egg and Rice Waste as Green Supercapacitor
Source: Molecules. 2023 Sep 9;28(18):6543. doi: 10.3390/molecules28186543 (PMC10536363; doi:10.3390/molecules28186543)
Supplement: Supplementary file 1 [file molecules-28-06543-s001.zip › molecules-2594381-supplementary.pdf]

Supplementary materials

In situ N, O co-doped nanoporous carbon derived from mixed egg and rice waste as green supercapacitor

Shumeng Qin [1], Peiliang Liu [1], Jieni Wang[2,1], Chenxiao Liu[2,1], Shuqin Zhang[2,1], Yijun Tian[2,1], Fangfang Zhang[2,1], Lin Wang[1], Leichang Cao[2,1*], Jinglai Zhang[2], Shicheng Zhang[3]

[1] Miami College, Henan University, Kaifeng 475004, China

[2] College of Chemistry and Molecular Sciences, Henan University, Kaifeng 475004, China

[3] Shanghai Key Laboratory of Atmospheric Particle Pollution and Prevention (LAP3), Department of Environmental Science and Engineering, Fudan University, Shanghai, 200433, China

[*] Corresponding Author: clch666@henu.edu.cn

Number of Pages: 4
Number of Figures: 3

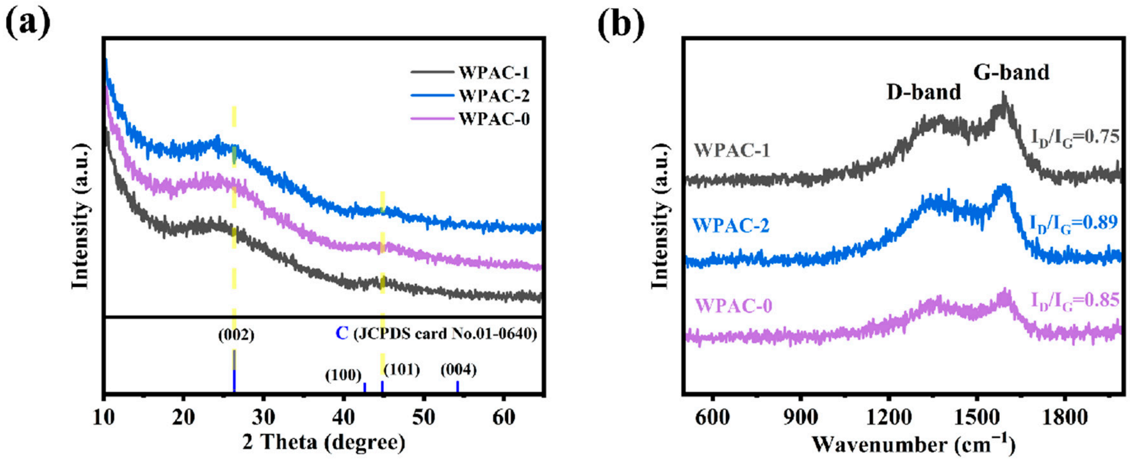

Figure S1. (a) XRD pattern and (b) Raman spectra of WPAC-0, WPAC-1, and WPAC-2.

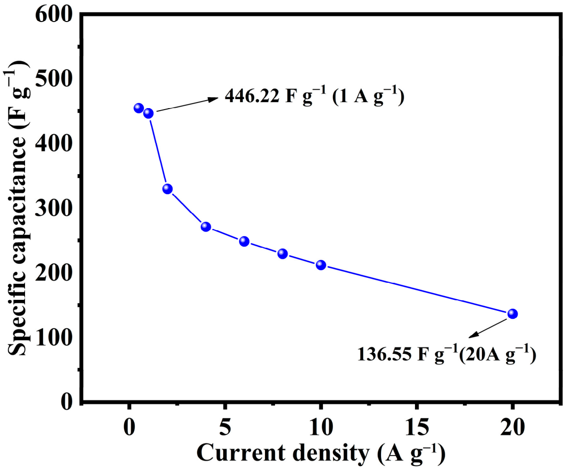

Figure S2. The specific capacitance of YPAC-1 at 0.5–20 A g^{-1}.

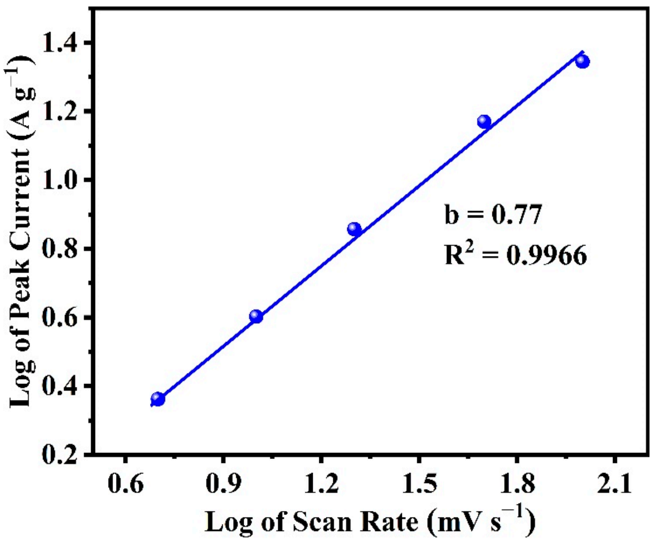

Figure S3. Relationship between peak current and scan rates at scan rates of 5–100 mV s^{-1}.
